# Adrenergic stimulation of adiponectin secretion in visceral mouse adipocytes is blunted in high-fat diet induced obesity

**DOI:** 10.1038/s41598-019-47113-8

**Published:** 2019-07-23

**Authors:** Saliha Musovic, Charlotta S. Olofsson

**Affiliations:** Department of Physiology/Metabolic Physiology, Institute of Neuroscience and Physiology, The Sahlgrenska Academy at University of Gothenburg, Medicinaregatan 11, SE-405 30 Göteborg, Sweden

**Keywords:** Type 2 diabetes, Exocytosis, Cell signalling, Type 2 diabetes

## Abstract

The hormone adiponectin is secreted by white adipocytes and has been put forward as a key mediator of obesity-linked insulin resistance and the metabolic syndrome. Although adiponectin was discovered two decades ago, the knowledge about the molecular and cellular regulation of its secretion is incomplete. Here we have investigated the adrenergic regulation of adiponectin secretion in primary visceral (gonadal) adipocytes isolated from lean or obese/diabetic mice. We show that visceral adipocyte adiponectin release is triggered by cAMP/catecholamines via signalling pathways involving adrenergic beta-3-receptors (β_3_ARs) and *E*xchange *P*rotein directly *A*ctivated by *c*AMP, isoform 1 (Epac1). The adrenergically stimulated adiponectin secretion is blunted in visceral adipocytes isolated from obese and diabetic mice and our results suggest the existence of a secretory defect. We have previously shown that adiponectin secretion in subcutaneous adipocytes is abolished in the obese/diabetic state due to reduced abundance of β_3_ARs and Epac1. However, here we show that protein levels of β_3_ARs and Epac1 are maintained in visceral adipocytes from obese/diabetic mice proposing that other molecular defects underlie the blunted adiponectin release. Gene expression analysis indicate diabesity-associated disturbances of the signalling downstream of Epac1 and/or the exocytotic process itself. Our study proposes that visceral adipocytes partake in the regulated secretion of adiponectin and may thus influence circulating levels of the hormone, in health and in metabolic disease.

## Introduction

The hormone adiponectin, secreted by white adipocytes, has been proposed as an important mediator of the crosstalk between the adipose tissue and other organs involved in regulation of whole body metabolism. A disturbance of this communication, due to disruptions at the adiponectin receptor level or a reduction of circulating adiponectin, leads to faulty lipid and glucose metabolism and has been linked to type 2 diabetes^1–3^. White adipose tissue (WAT) can be divided into two main compartments in the body, subcutaneous adipose tissue (SAT) and visceral adipose tissue (VAT), which differ both morphologically and with regard to metabolic and endocrine properties^4–6^. Whereas SAT is superficially located, VAT is situated in the abdominal cavity surrounding the internal organs. VAT has been shown to have a higher lipolytic activity compared to SAT, due to a greater abundance of beta-adrenergic receptors (βARs) in combination with a lower sensitivity to the anti-lipolytic pancreatic hormone insulin^7,8^. Several studies have reported a correlation between adiponectin and different adipose tissue depots^9–11^. Described adipose depot-related differences with regards to both adiponectin gene expression^12^ and synthesis/secretion^13^ suggest that chiefly subcutaneous adipose tissue is important for the maintenance of circulating adiponectin levels. Adiponectin levels are reduced in type 2 diabetes and serum adiponectin is negatively related to increased visceral adiposity^9–11^. Moreover, individuals with increased VAT are at greater risk of developing obesity-related disorders, such as type 2 diabetes and cardiovascular disease. This is likely in part due to the anatomical position of VAT, with direct portal drainage to the liver through the portal vein;^14^ free fatty acids and hormones released from VAT can directly affect glucose and liver metabolism.

The adiponectin release over extended time periods has been rather thoroughly investigated^15–19^. However, only few investigations have focused on the short-term (30–60 min) regulation of adiponectin secretion/exocytosis and adiponectin release has been shown to be induced by insulin^20,21^ and to require Ca^2+^-dependent signalling^20^. Own studies have investigated the regulation of adiponectin exocytosis in more detail, using a combination of electrophysiological and biochemical methods. We have demonstrated that catecholamines stimulate acute adiponectin release from subcutaneous adipocytes (isolated from inguinal white adipose tissue; IWAT) via activation of adrenergic beta-3-receptors (β_3_ARs) and *E*xchange *P*rotein directly *A*ctivated by *c*AMP, isoform 1 (Epac1). Using high-fat diet (HFD)-fed mice, we further showed that the adrenergic stimulation was abolished in obesity/type 2 diabetes due to reduced abundance of both β_3_ARs and Epac1^22^. We have also verified the cAMP/Epac-dependent stimulation of adiponectin release in human subcutaneous adipocytes^23^.

The objective of the current study was to investigate the pathophysiological regulation of short-term adiponectin secretion in visceral adipocytes. Although adiponectin has been suggested to be released from visceral adipose tissue depots (perivascular adipocytes), via β_3_AR-dependent pathways and to act as a vasorelaxant factor^24^, the detailed regulation of visceral adipocyte adiponectin secretion has never been investigated. In light of the depot-dependent differential functionality of adipose tissue^4–6^, it is essential to define how visceral adipocyte adiponectin secretion is regulated at a cellular and molecular level. By use of mouse gonadal white adipose tissue (GWAT) we here show that visceral adipocytes secrete adiponectin in response to catecholamines and that β_3_ARs and Epac1 are central to this regulation. The stimulated adiponectin secretion is blunted in diabesity but the mechanisms underlying the abrogated release differ from those characterised in IWAT adipocytes.

## Results

### Adrenergic stimulation of adiponectin secretion in primary visceral mouse adipocytes is Epac-dependent

**Figure 1:**
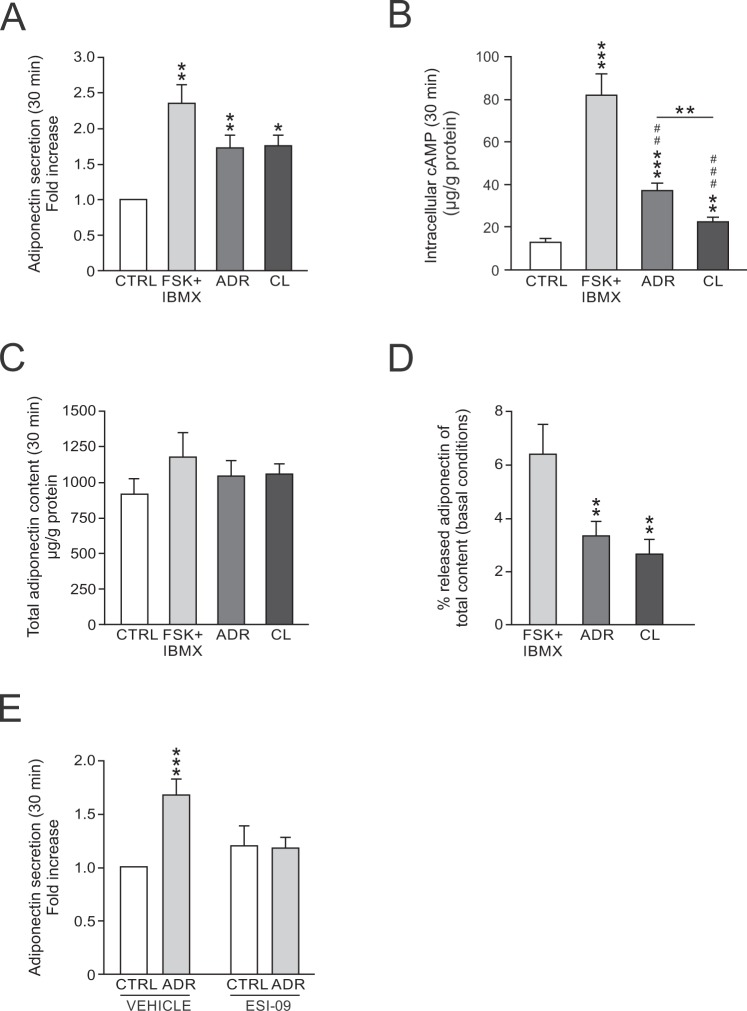
cAMP/adrenergic stimulation of adiponectin secretion in GWAT adipocytes. (**A**) Adiponectin secretion expressed as fold change compared to control (basal release) during 30 min treatment with forskolin (10 μM) together with IBMX (200 μM; FSK/IBMX), adrenaline (5 μM; ADR) or CL (1 μM). (**B**) Intracellular cAMP levels in stimulated primary adipocytes. (**C**) Total adiponectin content in stimulated primary GWAT adipocytes. (**D**) Percentage secreted adiponectin (over basal) of total adipocyte adiponectin content under basal (unstimulated) conditions. (**E**) Adrenaline-stimulated adiponectin release in adipocytes pre-treated with Epac-antagonist ESI-09 (10 μM). Results in (**A**–**C**) and (**E**) represent data from 4–8 mice. *P < 0.05; **P < 0.01, ***P < 0.001 vs. control. In **B**, symbols represent ^##^*P* < 0.01, ^###^*P* < 0.001 vs. FSK/IBMX. In **D**, results are from 4 mice. **P < 0.01, ***P < 0.001 vs. FSK/IBMX.

In order to study if visceral adipocyte short-term adiponectin secretion can be stimulated via cAMP/adrenergic signalling, mouse GWAT adipocytes were incubated with forskolin (FSK;10 µM) in combination with IBMX (200 µM; FSK/IBMX), adrenaline (ADR; 5 µM) or the β_3_AR agonist CL316243 (CL; 1 µM) respectively during 30 minutes. As presented in Fig. 1A, FSK/IBMX stimulated adiponectin secretion >2-fold over basal whereas the stimulatory effect of ADR and CL tended to be slightly weaker (P = 0.07 for ADR and 0.09 for CL vs. FSK/IBMX). The potent stimulation by FSK/IBMX is expected since this combination of compounds produces a large increase of intracellular cAMP (forskolin activates adenylyl cyclases and IBMX inhibits phosphodiesterases). Measurements of cAMP levels in cell homogenates showed that cAMP levels were ~5-fold increased in the FSK/IBMX group compared to control whereas the effect of ADR- or CL-treatment was smaller (Fig. 1B). Adiponectin was measured in cell lysates in order to confirm that the elevated release was due to stimulated secretion and not a result of increased adiponectin content. The adiponectin content was similar in cells exposed to either stimulatory agent (Fig. 1C). As shown in Fig. 1D, and in agreement with that found in IWAT adipocytes^22^, only a minute fraction (a maximum of ∼6% in FSK/IBMX-stimulated cells) of the total adiponectin was secreted during the incubations. The percentage released adiponectin was larger in response to FSK/IBMX compared to both ADR or CL.

We have previously shown that cAMP/catecholamine-triggered adiponectin release in IWAT adipocytes occurs via activation of Epac1^22^. To investigate the role of Epac in visceral adipocyte adiponectin secretion, GWAT cells were pre-treated with the Epac-antagonist ESI-09 (10 µM for 30 minutes) prior to incubation with ADR. ADR-stimulated adiponectin secretion was abolished in ESI-09 pre-treated cells (Fig. 1E).

**Figure 2:**
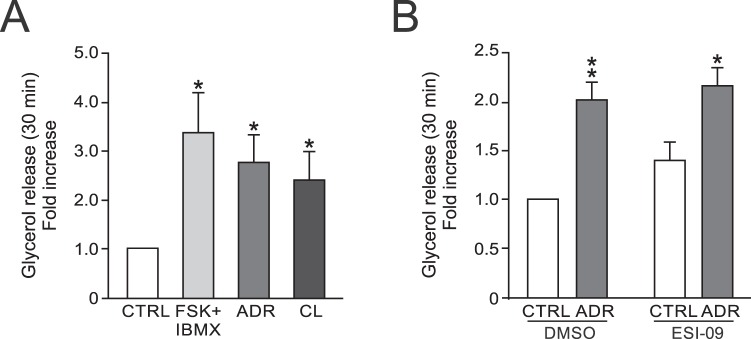
Lipolysis measured as glycerol release in GWAT adipocytes. (**A**) Glycerol release measured in samples from cells stimulated with FSK/IBMX, ADR or CL. (**B**) Lipolysis in cells incubated with ESI-09 prior to stimulation with adrenaline (ADR). Results in (**A**,**B**) are expressed as fold-change compared to control and represent 5–7 experiments. *P < 0.05; **P < 0.01, ***P < 0.001 vs. control. Glycerol release was measured in the same samples as analysed for adiponectin secretion in Fig. 1A and E.

Lipolysis is another adipocyte metabolic process that is stimulated via adrenergic/cAMP pathways, but that involves signalling via PKA^25^ and not Epac. As shown in Fig. 2A, lipolysis, measured as glycerol release, was increased in GWAT adipocytes following incubation with FSK/IBMX, ADR or CL. In agreement with the involvement of PKA-dependent pathways, glycerol release was unaffected by incubation with ESI-09 (Fig. 2B). Clearly, although adiponectin secretion is abrogated by the antagonist, the adipocytes retain their functionality. Interestingly, the Epac inhibitor tended to increase basal lipolysis (P = 0.1 vs. DMSO control).

### Impaired adiponectin secretion in visceral adipocytes isolated from obese/type 2 diabetic mice

**Figure 3:**
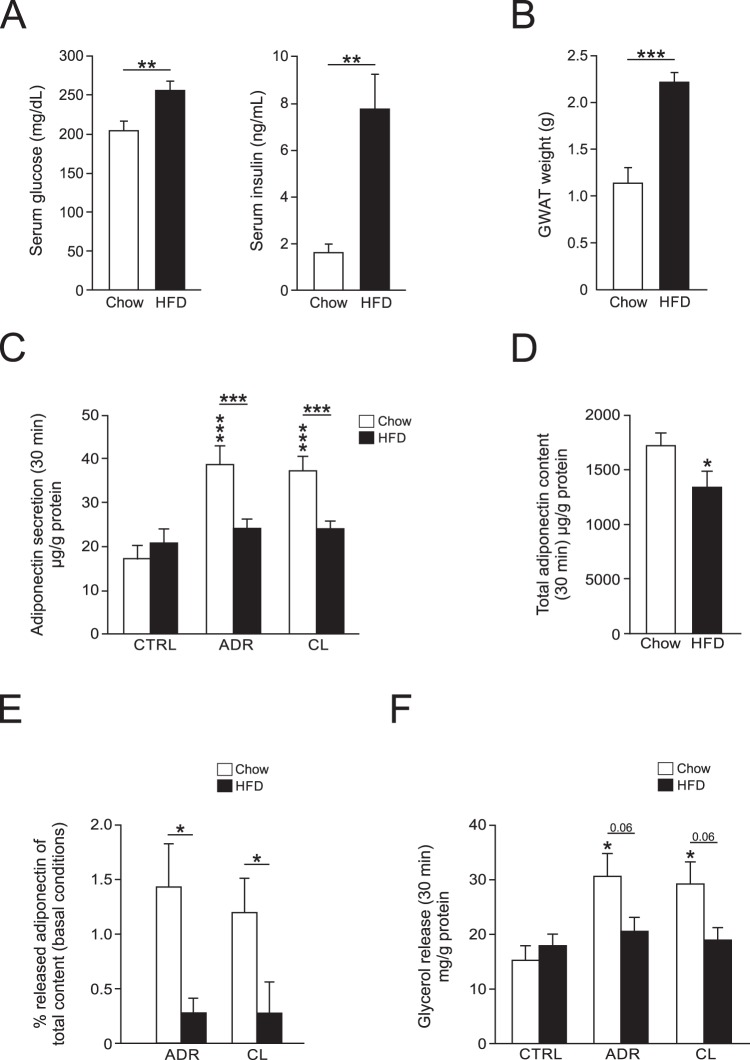
Adrenergic stimulation of adiponectin secretion in GWAT cells isolated from lean or obese/diabetic mice. (**A**) Serum glucose (left) and insulin (right) concentrations in mice fed chow or HFD for 8 weeks. (**B**) Weight of GWAT obtained from chow- or HFD-fed mice. (**C**) Adrenergically stimulated adiponectin secretion (30 min) in GWAT adipocytes isolated from chow- or HFD-fed mice. (**D**) Total adiponectin content in non-stimulated adipocytes isolated from chow- and HFD-fed mice. (**E**) Percentage secreted adiponectin (over basal) of total adipocyte adiponectin content under control conditions in chow- and HFD-adipocytes. (**F**) Glycerol release in chow- and HFD-adipocytes stimulated with ADR or CL (samples from experiments in **C**). Results in (**A**–**F**) are from 4–11 chow and 4–12 HFD mice. *P < 0.05; **P < 0.01, ***P < 0.001 vs. control.

We next studied short-term (30 min) adiponectin secretion in GWAT adipocytes from mice fed chow or HFD during eight weeks. HFD-fed mice were obese (average weight 42.4 ± 0.1 g compared to 30.9 ± 1.4 g for chow-fed animals) as well as diabetic, discernible as elevated serum glucose and insulin levels (Fig. 3A). The weight of GWAT isolated from obese/diabetic mice was 2-fold increased compared to GWAT from lean animals (Fig. 3B). Both ADR and CL elevated adiponectin secretion ~2.5-fold compared to control in GWAT adipocytes isolated from chow-fed mice. Adiponectin release triggered by ADR or CL was abolished in GWAT adipocytes isolated from obese mice while the basal (unstimulated) adiponectin release was unaffected by HFD-feeding (P = 0.4; Fig. 3C). Measurements in cell lysates showed that the adiponectin content was slightly reduced in HFD adipocytes compared to chow (Fig. 3D). Importantly, analysis of the percentage released adiponectin demonstrated that the fraction secreted from HFD adipocytes was significantly smaller compared to chow adipocytes (Fig. 3E). This finding demonstrates that the diabesity-associated abrogation of stimulated adiponectin release is not a result of the reduced content and underscores the existence of a secretory defect. As shown in Fig. 3F, adrenergically stimulated lipolysis was also blunted in HFD adipocytes.

### The cAMP signalling pathway remains intact in GWAT adipocytes isolated from obese/diabetic mice

**Figure 4:**
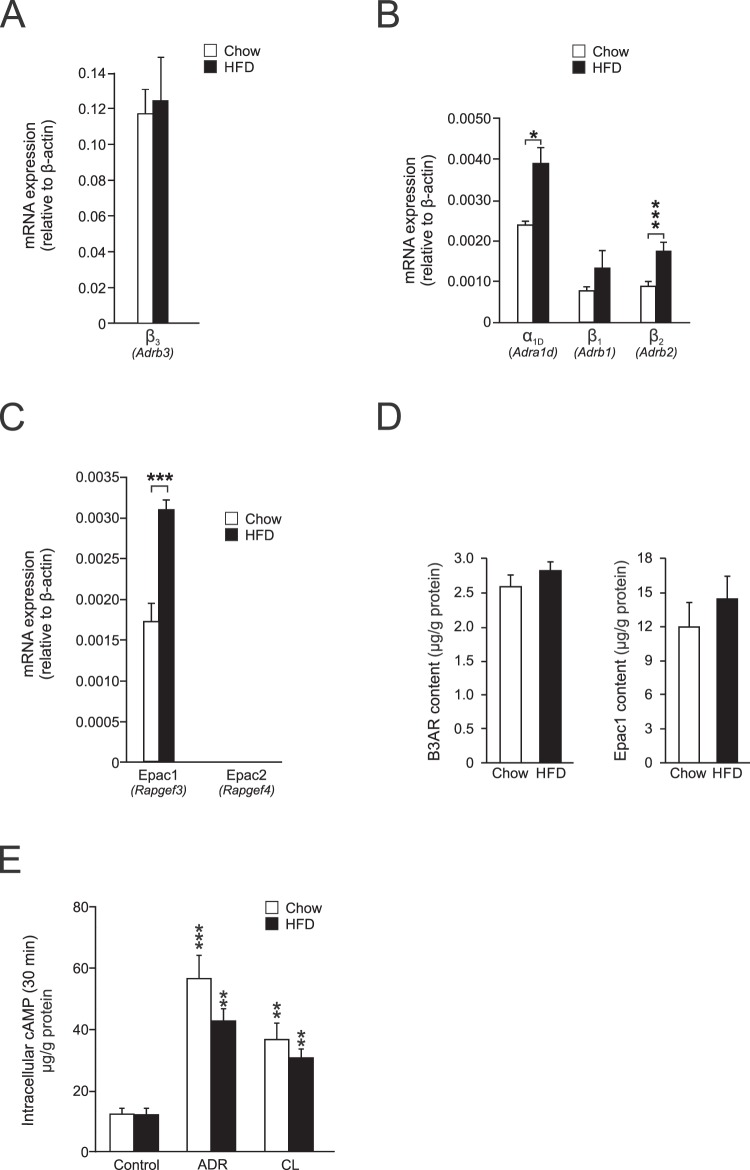
Expression of adrenergic receptors and Epac in GWAT as well as cAMP levels in adipocytes from lean and obese/diabetic mice. (**A**–**C**) Gene expression of adrenergic receptors and Epac-isoforms. Gene expression was normalized against β-actin (*Actb*) using the relative ΔC_t_ method. Primers were used at a conc of 500 nM. (**D**) Protein levels of β_3_AR receptor and Epac1 in GWAT adipocytes isolated from chow- and HFD-fed mice. (**E**) Intracellular cAMP levels in chow or HFD subcutaneous adipocytes during 30 min incubations in the presence of ADR or CL. Data in (**A**–**E**) are from 5 chow and 5 HFD mice. *P < 0.05; **P < 0.01, ***P < 0.001 vs. control.

Our previous studies using IWAT (subcutaneous) adipocytes show that the diabesity-associated abolishment of adrenergically stimulated adiponectin release largely results from reduced levels of β_3_ARs and Epac1^22^. To investigate if similar molecular defects could explain the diminished secretion of adiponectin in GWAT adipocytes from obese/diabetic mice, we measured the gene expression of ARs and Epac (isoform 1 and 2) in adipocytes from chow- and HFD-fed mice. As in IWAT^22^, the β_3_ARs (*Adrb3*) were amply expressed in GWAT adipocytes from chow-fed mice. In contrast to findings using IWAT fat cells, β_3_AR mRNA levels were unaltered in GWAT adipocytes isolated from HFD-fed mice (Fig. 4A). The β_1_ and β_2_ adrenergic receptors (*Adrb1* and *Adrb2*, respectively) were expressed at ~100-fold lower levels than β_3_ARs and the expression of β_2_ARs was upregulated in HFD adipocytes (Fig. 4B). Also α_1D_ARs (*Adra1d*) were expressed and the mRNA level was elevated in adipocytes from HFD-fed mice (Fig. 4B). In agreement with previous findings in adipocytes^22,26^, Epac1 was the isoform expressed and Epac2 could not be detected. The mRNA level of Epac1 was doubled in GWAT adipocytes from HFD-fed mice (Fig. 4C). Studies of protein expression of β_3_ARs and Epac1 yielded similar results (Fig. 4D), although Epac1 abundance was not significantly higher in HFD adipocytes compared to chow (P = 0.4). The high expression of β_3_ARs in GWAT adipocytes from HFD-fed mice indicates that ADR and CL potently triggers cAMP increases in these cells (although several other intracellular proteins, such as adenylyl cyclases and phospohdiesterases of course determine the cytoplasmic cAMP dynamics). As shown in Fig. 4E, intracellular cAMP levels indeed remained largely unaltered in GWAT adipocytes isolated from HFD-fed mice as compared to chow, both under control conditions and upon stimulation with ADR or CL. The ability of CL to elevate cAMP to a similar level in chow and HFD adipocytes is in agreement with the maintained abundance of β_3_ARs^22^.

In conclusion, the intact β_3_AR and Epac1 expression as well as the maintained CL-stimulated cAMP increase indicate that the diabesity-associated blunted adiponectin secretion in GWAT adipocytes is not due to signalling defects involving those proteins.

### Gene expression of proteins potentially involved in adiponectin synthesis and secretion

**Figure 5:**
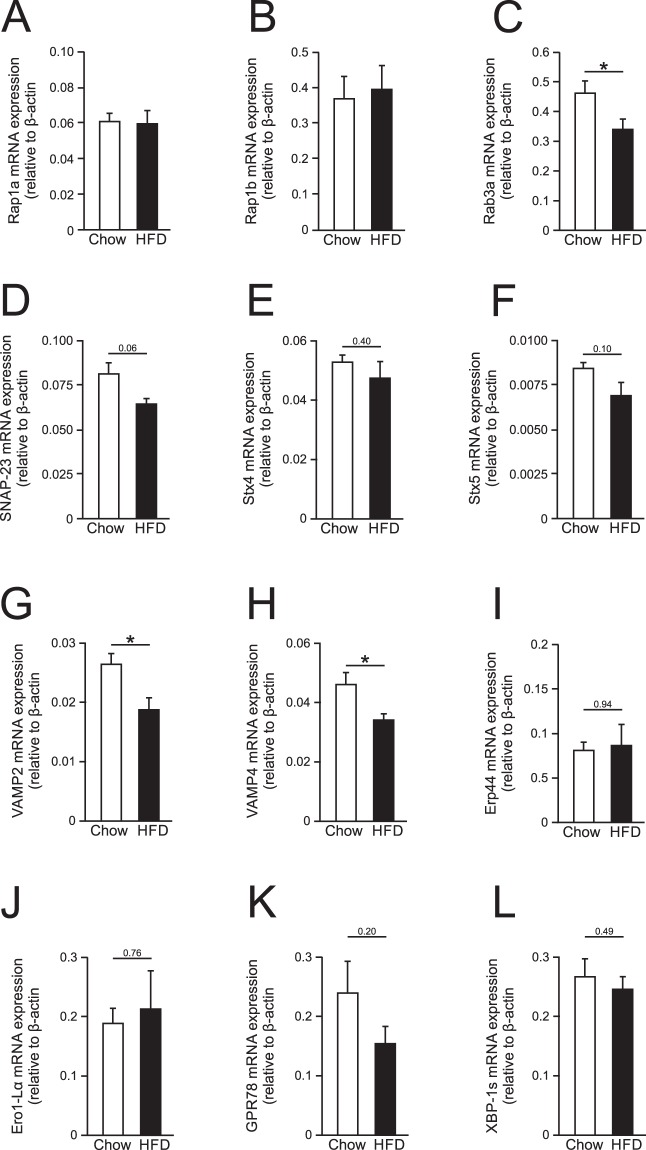
Gene expression of SNARE proteins, ER-stress markers and ER chaperones in GWAT adipocytes from lean and obese/diabetic mice. Gene expression of Rap1a (**A**), Rap1b (**B**), Rab3a (**C**), Snap-23 (**D**), Stx4 (**E**), Stx5 (**F**), Vamp2 (**G**), Vamp4 (**H**), Erp44 (**I**), Ero1α (**J**), GPR78 (**K**) and XBP-1s (**L**). Data in *A*-*L* are from 4–5 chow and 5 HFD mice. *P < 0.05 vs. control.

In search of mechanisms that could explain the blunted adiponectin secretion observed in GWAT adipocytes isolated form obese/diabetic mice, we investigated the gene expression of a number of molecular players hypothesised to be involved in regulating the catecholamine-triggered exocytosis. Epac proteins are guanine nucleotide exchange factors (GEFs) that signal via activation of small GTPases, in particular Rap1 that is a member of the Ras family^27–29^. The cAMP-induced exocytosis in endothelial cells has been shown to be regulated via an Epac-Rap1 signalling pathway^30^. In the sperm acrosome reaction, Epac, Rap1 and the small GTPase RAb3 have been demonstrated to act together to achieve exocytosis^31^. We measured mRNA levels of Rap1a, Rap1b and Rab3a (the Rab3 isoform expressed according to own micro array data) in GWAT adipocytes isolated from lean and obese animals. As shown in Fig. 5A–C, mRNA levels of Rap1a and Rap1b were unaffected by diet-induced obesity whereas expression of Rab3a was slightly reduced.

Hormone-containing vesicle plasma membrane fusion and release is typically mediated by the interaction of Soluble N-ethylmaleimide-sensitive factor activating protein receptor (SNARE) proteins that are located both on the secretory vesicles and on the plasma membrane^32^. We hypothesized that the blunted diabesity-associated adiponectin release in GWAT adipocytes is perhaps due to defects disturbing the adiponectin exocytosis process itself. To investigate this we measured mRNA levels of selected exocytotic proteins hypothesized to be involved in the adiponectin vesicle exocytosis process. It need to be emphasised that although a few proteins involved in the translocation of vesicles containing glucose transporter 4 (Glut4) in white adipocytes have been characterised^33–35^, those regulating adiponectin exocytosis are largely unknown. Thus, guided by the known involvement of different SNARE proteins in regulation of vesicle exocytosis^32^, findings in adipocytes^33–35^ and own microarray data, we decided to investigate SNAP-23, Syntaxin (Stx) 4 and 5 and vesicle-associated membrane protein (VAMP) 2 and 4. The expression of SNAP-23 and Stx5 tended to be slightly decreased in HFD adipocytes whereas Stx4 was unaffected (Fig. 5D–F). VAMP2 and VAMP4 mRNA levels were both reduced in adipocytes isolated from fat mice (Fig. 5G,H).

We further postulated that the adiponectin synthesis and/or assembly within the endoplasmic reticulum (ER) may be faulty in fat GWAT adipocytes, thus affecting the pool of releasable adiponectin. The folding of proteins within the endoplasmic reticulum (ER) ensues with the help of molecular chaperones^36^. The chaperones ERp44 and Ero1-Lalpha have been identified as key regulators of the ER-located posttranslational events allowing adiponectin to exit ER and to be secreted from adipocytes. The levels of both chaperones are tightly controlled in adipocytes and have been reported to be impacted by the cell metabolic state^37–39^. However, as shown in Fig. 5I and J, the GWAT adipocyte expression of ERp44 and Ero1-Lalpha was unaffected by HFD. A disrupt ER function causes mis- or unfolded adiponectin to accumulate within the ER and activates a process known as the unfolded protein response (UPR), aiming to solve the problem. Sustained over activation of UPR results in a condition termed ER stress^40^. Obese adipose tissue often displays chronic ER stress and UPR activation, a state that has been linked to altered adipokine release, chronic inflammation and insulin resistance^41,42^. Chronic ER stress has been associated with lowered circulating adiponectin levels^43^. To investigate if ER stress/UPR (resulting in accumulation of adiponectin within ER) could explain the blunted adiponectin secretion in GWAT adipocytes isolated from HFD-fed mice, we measured the expression of the UPR markers GPR78 (BiP) and XBP-1s. As shown in Fig. 5K and L, neither GPR78 nor XBP-1s mRNA levels were significantly altered in HFD adipocytes.

## Discussion

Although adiponectin was discovered now more than 20 years ago^44^, we have only scratched the surface regarding the molecular and cellular regulation of its secretion. White adipose tissue is an unusual endocrine organ; in contrast to other hormone-releasing tissues, such as the endocrine pancreas or adrenal medulla, fat tissue is located at numerous locations in the body. Subcutaneous fat is situated beneath the skin whereas visceral fat encloses our inner organs and the fat depots differ with regard to cellularity, vascularization, innervation, inflammation and functionality. Visceral adipose tissue has been described to be more sensitive to adrenergic stimulation and an excess of visceral fat is associated with metabolic disease and increased mortality^4–6^. Own previous work has defined that adiponectin vesicles in adipocytes of subcutaneous origin (primary human and mouse adipocytes as well as cultured 3T3-L1 adipocytes) are rapidly released in response to an elevation of cytoplasmic cAMP and that intracellular Ca^2+^ augments the secretion^23,45,46^. We have further demonstrated the catecholamine-triggered exocytosis of adiponectin vesicles and that catecholamine-stimulated adiponectin secretion is blunted in subcutaneous adipocytes from animals with diet-induced obesity. The abrogated release is due to decreased abundance of β_3_ARs and Epac1^22^.

In this work we show that the molecular regulation of adiponectin release in visceral (GWAT) adipocytes is in several aspects similar to that in subcutaneous fat cells: 1) GWAT adipocyte adiponectin secretion is likewise stimulated via adrenergic pathways involving β_3_ARs and Epac1; 2) The catecholamine/CL-stimulated secretion is much reduced in GWAT cells isolated from HFD-fed mice. However, the molecular alterations responsible for the blunted adiponectin release clearly differ from the catecholamine resistance described in subcutaneous fat cells^22^ as protein levels of β_3_ARs and Epac1 remain largely unaltered in GWAT adipocytes isolated from HFD-fed mice (Fig. 4D).

The adiponectin content in visceral HFD adipocytes is slightly (~20%) reduced compared to chow cells. As can be seen in Figs 1D and 3E, only a small fraction (~1–3% with ADR or CL) of the total cell adiponectin is secreted during the short-term stimulations carried out here. In other hormone releasing cell types, a large reduction (>75%) of hormone content is required to affect the number of releasable hormone-containing vesicles^47^. This is because only a small portion of the hormone contained within a secreting cell belongs to the releasable pool (the large bulk of the hormone is instead functionally located upstream of the exocytotic sites)^48^. In Fig. 3E we show that the fraction of released adiponectin, when compared to total content, is significantly lower in HFD than in chow adipocytes, despite the reduced adiponectin content. This provides convincing support for that a reduction of adiponectin content does not underlie the blunted catecholamine/CL-triggered adiponectin release. Collectively our findings propose that the abolished release is instead caused by a secretory disturbance.

The abrogated secretion can not be explained by altered abundance of adrenergic receptors other than β_3_ARs. As shown in Fig. 4B, β_2_ARs are upregulated as are α_1_ARs; this shift in gene expression, if translational to the protein level, would if anything stimulate adiponectin exocytosis (β_2_ARs elevates cAMP and α_1_ARs increases Ca^2+^, another important regulator of adiponectin vesicle exocytosis in subcutaneous fat cells^23^). Moreover, in subcutaneous white adipocytes, the contribution of other adrenergic receptors to adiponectin release appears to be minor^22^. Results in Figs 1A and 3C, showing that CL triggers adiponectin secretion as potently as ADR, indicates that catecholamines stimulate adiponectin secretion primarily via activation of β_3_ARs also in visceral adipocytes. The largely unaffected cAMP-levels in Fig. 4E strongly support that adrenergic signalling remains sufficiently intact to elevate cAMP in HFD adipocytes to a level similar to that in chow cells (although it should be noted that those measurements do not yield information about possible local alterations of cAMP at specific plasma membrane-near micro domains^49,50^). In contrast, results in^22^ show a decrease in cAMP in response to CL in IWAT adipocytes isolated from obese and diabetic mice, fat cells with disrupt β_3_AR signalling. In conclusion, neither protein expression nor the intracellular cAMP level indicate that the cAMP-generating pathway is disturbed in adipocytes from HFD-fed mice. Considering that cAMP levels are unaltered, it may appear puzzling that the lipolysis is abolished in HFD GWAT adipocytes (Fig. 3F). However, the abrogated lipolytic response may be due to downstream defects in the signalling pathway. For example, reduced gene expression and lower activity of the adipocyte hormone-sensitive lipase (HSL; the enzyme hydrolysing triglycerides to free fatty acids and glycerol) has been reported in diabetes^51,52^. Desensitization of β_3_ARs have been suggested to arise in obesity^53^ but other studies have shown that β_3_ARs are less prone to desensitization compared to β_1_ and β_2_ ARs^54,55^. Although we are unable to exclude the possibility that β_3_AR desensitization is involved in the observed blunted adiponectin secretion, the maintained intracellular cAMP levels in HFD adipocytes exposed to ADR or CL (Fig. 4E), strongly suggest that the signalling pathway is not disturbed at the receptor level.

Epac was identified as late as 1998 when this cAMP-guanine exchange factor was discovered to mediate the cAMP-dependent but PKA-independent activation of Rap1^28^. Epac is expressed in two isoforms (Epac1 and Epac2) that participate in the regulation of a large array of biological functions in multiple tissues. In neuronal and endocrine tissue, Epac controls a pathway of cAMP-reliant exocytosis that is not mediated via PKA (reviewed in^27^). In view of this, we hypothesized that the cAMP/Epac-triggered adiponectin exocytosis involves signalling via Rap1 and that reduced adipocyte Rap1 levels could underlie the blunted secretory response in GWAT adipocytes from obese/diabetic mice. In further support for a role of Rap1 in diabesity, mice ablated for Rap1 display a metabolically disturbed phenotype (exemplified by accumulation of visceral fat as well as elevated plasma inulin and glucose levels), suggesting the involvement of Rap1 in metabolic regulation^56^. However, data in Fig. 5 show that Rap1 mRNA levels were unaltered by the diet-induced obesity and that the gene expression of Rab3a (involved in the Epac-Rap1 signalling pathway^30^) was only slightly decreased.

SNARE proteins constitute a large family of proteins that mediate vesicle fusion with the plasma membrane in both endocrine and neuronal cells. Their function is regulated by variations of intracellular Ca^2+^, cAMP and ATP^32^, thus by mediators known to also control the regulated exocytosis of adiponectin^23^. The key SNARE involved in the formation of the exocytotic complex required for Ca^2+^-dependent exocytosis of neurotransmitters or hormones is Snap-25^32^. Snap-25 is however not expressed in non-neuroendocrine tissues (own micro array data confirm its absence in both IWAT and GWAT). In white adipose tissue, the homologue Snap-23 is instead present^35^ where it has been shown to regulate translocation of Glut4 to the plasma membrane. Snap-23 has been shown to, in a similar manner as Snap-25, interact with syntaxins and vesicle-associated membrane proteins (VAMPs)^33–35^. In our search to explain the secretory defect in visceral adipocytes from obese/diabetic mice, we detected reduced gene expression of VAMP2 and 4 whereas Snap-23 and Stx5 tended to be decreased (Fig. 5).

Other defects, upstream of the exocytotic machinery, may also lead to reduced stimulated adiponectin release. Faulty adiponectin synthesis and/or post-translational modification could result in trapping or degradation of adiponectin within ER^37–39,43^ and also be manifested as blunted secretion. However, the finding that gene expression of the chaperones ERp44 and Ero1-Lalpha (involved in ER-located posttranslational modifications of adiponectin) and the ER stress/UPR markers XBP-1s and GPR78 were unaltered in HFD adipocytes, suggests that faulty adiponectin synthesis or ER-associated trafficking is unlikely to cause the reduced secretion.

In conclusion, visceral (GWAT) adipocyte adiponectin secretion is clearly triggered via adrenergic signalling pathways involving β_3_ARs and Epac1. A pronounced decrease of catecholamine/CL-stimulated adiponectin release is evident in adipocytes isolated from obese/diabetic mice, but the blunted secretion appears to be due to defects that differ from those defined in subcutaneous (IWAT) HFD adipocytes (lower abundance of β_3_ARs and Epac1;^22^). The finding that diet-induced obesity affects the adiponectin signalling pathway differently in IWAT and GWAT adipocytes is perhaps not as surprising as it may first appear considering that visceral and subcutaneous fat have diverse origin^57^ and several shown functional differences^4–11^. Our results indicate that the signalling downstream of Epac and/or the exocytotic process itself may be defect in the obese/diabetic state. The here shown gene expression data display decreases of mRNA levels for a few investigated proteins but the reduction is modest and it is questionable if such small alterations are on their own sufficient to affect adiponectin exocytosis. However, it is possible that a slightly reduced abundance of several proteins involved in the regulation of adiponectin exocytosis, jointly leads to defect secretion of the adipokine. It needs to be acknowledged that a multitude of exocytotic proteins exists and the role of specific SNAREs for adiponectin exocytosis is to this point unknown. Thus, mediators and proteins downstream of Epac that affect the adiponectin secretory process need to be defined and their role for the diabesity-induced blunted adiponectin exocytosis must be studied in greater detail, both in subcutaneous and in visceral adipocytes. None the less, we believe that the data presented here signpost that visceral adipocytes are important for the regulation of adiponectin release and thus for circulating levels of the hormone, in health and in metabolic disease^57^.

## Methods

### Animal work

**Figure 6:**
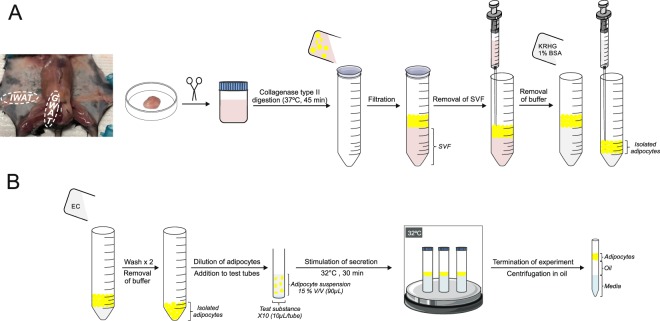
Schematic illustration of GWAT adipocytes isolation and incubation. (**A**) Graphic of the adipocyte isolation procedure including photo showing the localisation of the gonadal white adipose tissue (GWAT) depot in relation to inguinal (IWAT). (**B**) Graphic illustration of how the incubations for stimulation of adiponectin secretion are carried out. See text for more details. This figure was made using Servier Medical Art templates, licensed under the Creative Commons Attribution 3.0 Unported License; https://smart.servier.com.

Gonadal adipose tissue (GWAT) was isolated from 10–15 weeks old male C57BL/6 J mice (KRHG; 3% BSA). Adipose tissue was minced and digested using Collagenase type II (1 mg/mL in KRHG; 3% BSA, 45 min; 37 °C). After incubation with collagenase, the adipocyte suspension was poured through a 100 µm nylon mesh. Adipocytes floating on top were washed twice with KRHG buffer (1% BSA) and immediately used for experiments or snap-frozen in liquid nitrogen and kept at -80 °C. A graphic summary showing adipose tissue location and cell isolation is shown in Fig. 6A.

5-week old male mice were fed regular chow (Global Diet #2016, Harlan-Teklad) or high fat diet (60% kcal from fat; D12492, Research Diets Inc.) during 8 weeks. Animal work was approved by the Regional Ethical Review Board in Gothenburg.

### Adiponectin secretion and levels in isolated primary adipocytes

Isolated primary adipocytes were diluted to 15% V/V in 5 mM glucose extra cellular (EC) solution containing indicated test substances and incubated for 30 minutes at 32 °C under gentle shaking conditions. Primary adipocyte incubations were terminated by centrifugation in diisonyl phthalate (Sigma-Aldrich) followed by snap freezing in dry ice. Tubes were cut through the oil layer at two points, separating cells from media. EC aliquots and cell homogenates were stored at −80 °C. Secreted adiponectin (measured with mouse ELISA DuoSets; R&D Systems) was expressed in relation to total protein content (Bradford protein assay). A graphic summary of adipocyte incubation for stimulation of adiponectin secretion is shown in Fig. 6B. The EC solution contained (in mM) 140 NaCl, 3.6 KCl, 2 NaHCO_3_, 0.5 NaH_2_PO_4_, 0.5 MgSO_4_, 5 HEPES (pH 7.4 with NaOH), 2.6 CaCl_2_, and 5 glucose.

### Serum glucose, insulin and adiponectin levels

Animals were fasted during 4 hours prior to termination. Blood was collected and serum glucose levels were measured with glucose meter (Bayer Contour XT). The insulin concentration was analyzed using ELISA Mouse insulin kit (No. 10-1247-01; Mercodia).

### Lipolysis and intracellular cAMP measurements

Glycerol released into media was measured with free glycerol assay according to manufacturer protocol (G7793 and F6428, Sigma-Aldrich). Intracellular cAMP levels were measured in cell lysate with Cyclic AMP XP Assay Kit (No. 4339; Cell Signaling).

### Quantitative Real-Time PCR

RNA from isolated primary adipocytes was isolated and purified with TRIzol (Life Technologies) and ReliaPrep™ RNA Cell Miniprep System (Promega). Total RNA was measured and converted to equal amount cDNA (5 ng/µL) by qScript Flex cDNA Kit (Quanta Biosciences). SYBR Select Master Mix (Life Technologies) was used for quantitative RT-PCR. Gene expression of genes of interest was normalized against β-actin (*Actb*) using the relative ΔC_t_ method (for primer sequences, see Supplementary Table 1). Primers were used at a concentration of 500 nM.

### Protein measurements

Abundance of β_3_ARs and Epac 1 protein levels were measured with mouse specific ELISA (MBS2705329, MBS9329634, My Bio Source).

All methods and experimental work were conducted in adherence with institutional regulations and guidelines.

### Data analysis

The statistical of significance of variance between two means was calculated with Student’s t-test or ANOVA when appropriate. All data are presented as mean values ± SEM for designated number of experiments.

## Supplementary information

Supplementary Table 1

## Data Availability

The data supporting the findings of this study are available within the article.

## References

[1] Wang ZV, Scherer PE (2016). Adiponectin, the past two decades. J Mol Cell Biol.

[2] Ouchi N, Parker JL, Lugus JJ, Walsh K (2011). Adipokines in inflammation and metabolic disease. Nature Reviews Immunology.

[3] Yamauchi T (2001). The fat-derived hormone adiponectin reverses insulin resistance associated with both lipoatrophy and obesity. Nature medicine.

[4] Ibrahim MM (2010). Subcutaneous and visceral adipose tissue: structural and functional differences. Obes Rev.

[5] Wajchenberg BL (2000). Subcutaneous and visceral adipose tissue: their relation to the metabolic syndrome. Endocr Rev.

[6] Hamdy O, Porramatikul S, Al-Ozairi E (2006). Metabolic obesity: the paradox between visceral and subcutaneous fat. Curr Diabetes Rev.

[7] Arner P, Hellstrom L, Wahrenberg H, Bronnegard M (1990). Beta-adrenoceptor expression in human fat cells from different regions. J Clin Invest.

[8] Bolinder J, Kager L, Ostman J, Arner P (1983). Differences at the receptor and postreceptor levels between human omental and subcutaneous adipose tissue in the action of insulin on lipolysis. Diabetes.

[9] Gavrila A (2003). Serum adiponectin levels are inversely associated with overall and central fat distribution but are not directly regulated by acute fasting or leptin administration in humans: cross-sectional and interventional studies. J Clin Endocrinol Metab.

[10] Lihn AS (2004). Lower expression of adiponectin mRNA in visceral adipose tissue in lean and obese subjects. Mol Cell Endocrinol.

[11] Cote M (2005). Adiponectinemia in visceral obesity: impact on glucose tolerance and plasma lipoprotein and lipid levels in men. J Clin Endocrinol Metab.

[12] Fisher FM (2002). Differences in adiponectin protein expression: effect of fat depots and type 2 diabetic status. Hormone and metabolic research=Hormon- und Stoffwechselforschung=Hormones et metabolisme.

[13] Meyer LK, Ciaraldi TP, Henry RR, Wittgrove AC, Phillips SA (2013). Adipose tissue depot and cell size dependency of adiponectin synthesis and secretion in human obesity. Adipocyte.

[14] Rytka JM, Wueest S, Schoenle EJ, Konrad D (2011). The portal theory supported by venous drainage-selective fat transplantation. Diabetes.

[15] Blumer RM (2008). Regulation of adiponectin secretion by insulin and amino acids in 3T3-L1 adipocytes. Metabolism.

[16] Cong L (2007). Regulation of adiponectin and leptin secretion and expression by insulin through a PI3K-PDE3B dependent mechanism in rat primary adipocytes. Biochem J.

[17] Fasshauer M (2003). Adiponectin gene expression and secretion is inhibited by interleukin-6 in 3T3-L1 adipocytes. Biochem Biophys Res Commun.

[18] Pereira RI, Draznin B (2005). Inhibition of the phosphatidylinositol 3′-kinase signaling pathway leads to decreased insulin-stimulated adiponectin secretion from 3T3-L1 adipocytes. Metabolism.

[19] Xie L, O’Reilly CP, Chapes SK, Mora S (2008). Adiponectin and leptin are secreted through distinct trafficking pathways in adipocytes. Biochim Biophys Acta.

[20] Bogan JS, Lodish HF (1999). Two compartments for insulin-stimulated exocytosis in 3T3-L1 adipocytes defined by endogenous ACRP30 and GLUT4. J Cell Biol.

[21] Lim CY, Hong W, Han W (2015). Adiponectin is released via a unique regulated exocytosis pathway from a pre-formed vesicle pool on insulin stimulation. Biochem J.

[22] Komai AM (2016). White Adipocyte Adiponectin Exocytosis Is Stimulated via beta3-Adrenergic Signaling and Activation of Epac1: Catecholamine Resistance in Obesity and Type 2 Diabetes. Diabetes.

[23] Komai AM, Brannmark C, Musovic S, Olofsson CS (2014). PKA-independent cAMP stimulation of white adipocyte exocytosis and adipokine secretion: modulations by Ca2+ and ATP. J Physiol.

[24] Saxton SN (2018). Role of Sympathetic Nerves and Adipocyte Catecholamine Uptake in the Vasorelaxant Function of Perivascular Adipose Tissue. Arterioscler Thromb Vasc Biol.

[25] Fruhbeck G, Mendez-Gimenez L, Fernandez-Formoso JA, Fernandez S, Rodriguez A (2014). Regulation of adipocyte lipolysis. Nutrition research reviews.

[26] Petersen RK (2008). Cyclic AMP (cAMP)-mediated stimulation of adipocyte differentiation requires the synergistic action of Epac- and cAMP-dependent protein kinase-dependent processes. Mol Cell Biol.

[27] Schmidt M, Dekker FJ, Maarsingh H (2013). Exchange protein directly activated by cAMP (epac): a multidomain cAMP mediator in the regulation of diverse biological functions. Pharmacological reviews.

[28] de Rooij J (1998). Epac is a Rap1 guanine-nucleotide-exchange factor directly activated by cyclic AMP. Nature.

[29] Kawasaki H (1998). A family of cAMP-binding proteins that directly activate Rap1. Science.

[30] van Hooren KW (2012). The Epac-Rap1 signaling pathway controls cAMP-mediated exocytosis of Weibel-Palade bodies in endothelial cells. J Biol Chem.

[31] Branham MT (2009). Epac activates the small G proteins Rap1 and Rab3A to achieve exocytosis. J Biol Chem.

[32] Kasai H, Takahashi N, Tokumaru H (2012). Distinct initial SNARE configurations underlying the diversity of exocytosis. Physiol Rev.

[33] Kawanishi M (2000). Role of SNAP23 in insulin-induced translocation of GLUT4 in 3T3-L1 adipocytes. Mediation of complex formation between syntaxin4 and VAMP2. J Biol Chem.

[34] Olson AL, Knight JB, Pessin JE (1997). Syntaxin 4, VAMP2, and/or VAMP3/cellubrevin are functional target membrane and vesicle SNAP receptors for insulin-stimulated GLUT4 translocation in adipocytes. Mol Cell Biol.

[35] St-Denis JF, Cabaniols JP, Cushman SW, Roche PA (1999). SNAP-23 participates in SNARE complex assembly in rat adipose cells. Biochem J.

[36] McLaughlin M, Vandenbroeck K (2011). The endoplasmic reticulum protein folding factory and its chaperones: new targets for drug discovery?. British journal of pharmacology.

[37] Hampe L (2015). Regulation and Quality Control of Adiponectin Assembly by Endoplasmic Reticulum Chaperone ERp44. J Biol Chem.

[38] Phillips SA (2009). Selective regulation of cellular and secreted multimeric adiponectin by antidiabetic therapies in humans. Am J Physiol Endocrinol Metab.

[39] Wang ZV (2007). Secretion of the adipocyte-specific secretory protein adiponectin critically depends on thiol-mediated protein retention. Mol Cell Biol.

[40] Lindholm D, Korhonen L, Eriksson O, Koks S (2017). Recent Insights into the Role of Unfolded Protein Response in ER Stress in Health and Disease. Front Cell Dev Biol.

[41] Lee J, Ozcan U (2014). Unfolded protein response signaling and metabolic diseases. J Biol Chem.

[42] Kawasaki N, Asada R, Saito A, Kanemoto S, Imaizumi K (2012). Obesity-induced endoplasmic reticulum stress causes chronic inflammation in adipose tissue. Sci Rep.

[43] Zhou L (2010). DsbA-L alleviates endoplasmic reticulum stress-induced adiponectin downregulation. Diabetes.

[44] Scherer PE, Williams S, Fogliano M, Baldini G, Lodish HF (1995). A novel serum protein similar to C1q, produced exclusively in adipocytes. J Biol Chem.

[45] Brännmark Cecilia, Lövfors William, Komai Ali M., Axelsson Tom, El Hachmane Mickaël F., Musovic Saliha, Paul Alexandra, Nyman Elin, Olofsson Charlotta S. (2017). Mathematical modeling of white adipocyte exocytosis predicts adiponectin secretion and quantifies the rates of vesicle exo- and endocytosis. Journal of Biological Chemistry.

[46] El Hachmane MF, Komai AM, Olofsson CS (2015). Cooling Reduces cAMP-Stimulated Exocytosis and Adiponectin Secretion at a Ca2+-Dependent Step in 3T3-L1 Adipocytes. PLoS One.

[47] Olofsson CS (2007). Long-term exposure to glucose and lipids inhibits glucose-induced insulin secretion downstream of granule fusion with plasma membrane. Diabetes.

[48] Olofsson CS (2002). Fast insulin secretion reflects exocytosis of docked granules in mouse pancreatic B-cells. Pflugers Arch.

[49] Rybin VO, Xu X, Lisanti MP, Steinberg SF (2000). Differential targeting of beta -adrenergic receptor subtypes and adenylyl cyclase to cardiomyocyte caveolae. A mechanism to functionally regulate the cAMP signaling pathway. J Biol Chem.

[50] Willoughby D (2012). Organization of cAMP signalling microdomains for optimal regulation by Ca2+ entry. Biochemical Society transactions.

[51] Watt MJ (2005). Hormone-sensitive lipase is reduced in the adipose tissue of patients with type 2 diabetes mellitus: influence of IL-6 infusion. Diabetologia.

[52] Jocken JW (2007). Adipose triglyceride lipase and hormone-sensitive lipase protein expression is decreased in the obese insulin-resistant state. J Clin Endocrinol Metab.

[53] Withers SB (2014). Mechanisms of adiponectin-associated perivascular function in vascular disease. Arterioscler Thromb Vasc Biol.

[54] Masuo K, Lambert GW (2011). Relationships of adrenoceptor polymorphisms with obesity. J Obes.

[55] Schena Giorgia, Caplan Michael J. (2019). Everything You Always Wanted to Know about β3-AR * (* But Were Afraid to Ask). Cells.

[56] Martinez P (2013). RAP1 protects from obesity through its extratelomeric role regulating gene expression. Cell Rep.

[57] Chau YY (2014). Visceral and subcutaneous fat have different origins and evidence supports a mesothelial source. Nat Cell Biol.

