# Supplementary material for: Adrenergic stimulation of adiponectin secretion in visceral mouse adipocytes is blunted in high-fat diet induced obesity
Source: Sci Rep. 2019 Jul 23;9:10680. doi: 10.1038/s41598-019-47113-8 (PMC6650418; doi:10.1038/s41598-019-47113-8)
Supplement: Supplementary file 1 — Supplementary Table 1 [file 41598_2019_47113_MOESM1_ESM.pdf]

Adrenergic stimulation of adiponectin secretion in visceral mouse adipocytes is blunted in high-fat diet induced obesity

Saliha Musovic, Charlotta S Olofsson

Department of Physiology/Metabolic Physiology, Institute of Neuroscience and Physiology, The Sahlgrenska Academy at University of Gothenburg, Medicinaregatan 11, SE-405 30 Göteborg, Sweden

Running title: *Adiponectin secretion in visceral adipocytes*

Word count: 4380

Corresponding author:

Dr Charlotta Olofsson

University of Gothenburg, The Sahlgrenska Academy

Dept. Neuroscience and Physiology, Section of Metabolic Physiology

Postal adress: Box 432

SE-405 30 Göteborg, Sweden

ORCID ID: 0000-0001-8824-3151

E-mail: charlotta.olofsson@gu.se

Supplementary Table 1

Alias	Gene symbol	Forward primer 5´-3´	Reverse primer 3´-5´
β-actin	*Actb*	GACCCAGATCATGTTTGAGA	GAGCATAGCCCTCGTAGAT
α_{1D}	*Adra1d*	AGTGGGTGTCTTCCTAGCC	GCCTAGAACCTCCATAGTGGC
β_1	*Adrb1*	CTCATCGTGGTGGGTAACGTG	ACACACAGCACATCTACCGAA
β_2	*Adrb2*	GGGAACGACAGCGACTTCTT	GCCAGGACGATAACCGACAT
β_3	*Adrb3*	GGCCCTCTCTAGTTCCCAG	TAGCCATCAAACCTGTTGAGC
Epac1	*Rapgef3*	TCTTACCAGCTAGTGTTCGAGC	AARGCCGATATAGTCGCAGATG
Epac2	*Rapgef4*	CAAGGAGAATGTCCCTTCAGAGA	GAGCATAGCCCTCGTAGAT
SNAP-23	*SNAP23*	AATCCTGGGTTTAGCCATTGAGTC	TTGGTCCATGCCTTCTTCTATGC
Stx4	*STX4*	CCCGGACGACGAGTTCTTC	TTTGATCTCCTCTCGCAGGTT
Stx5	*STX5*	CAAACAAGCAAACCGGCTCTG	ATCTTTCCCAATGCGCCTGG
VAMP2	*VAMP2*	CACAATCTGGTTCTTTGAGGAG	AGAGACTTCAGGCAGGAATTAG
VAMP4	*VAMP4*	TTCAAGCGCCACCTAAATGAT	CCAGATGGTCCCCGTAGAAAAA
XBP-1s	*XBP1*	GGTCTGCTGAGTCCGCAGCAGG	GAAAGGGAGGCTGGTAAGGAAC
BiP	*HSPA5*	CCTCTCTGGTGATCAGGATA	CGTGGAGAAGATCTGAGACT
ERp44	*ERP44*	TGCGGTCTTCCTGTCTTTAGC	AACGACACCAGTCAGCATAAAA
Ero1-Lalpha	*ERO1A*	TTCTGCCAGGTTAGTGGTTACC	GTTTGACGGCACAGTCTCTTC
Rap1a	*Rap1a*	ATGCGTGAGTACAAGCTAGTAGT	AATCTACCTCGACTTGCTTTCTG
Rap1b	*Rap1b*	ATGCGTGAATATAAGCTCGTCG	GCGAAGCCTTGTCCGTTCT
Rab3a	*Rab3a*	GTGGGCAAAACCTCGTTCCT	TCCTCTTGTCGTTGCGGTAGA
